# Satellite observations indicate that chia uses less water than other crops in warm climates

**DOI:** 10.1038/s42003-024-06841-y

**Published:** 2024-09-30

**Authors:** Brian Kirsch, Joshua B. Fisher, Thomas Piechota, Mohammad Hassani, Diego C. Suardiaz, Radhika Puri, Joseph Cahill, Hagop S. Atamian

**Affiliations:** 1 Schmid College of Science and Technology, Chapman University, Orange, CA USA; 2 Fowler School of Engineering, Chapman University, Orange, CA USA; 3 Faculty of Biological Sciences, University of Veracruz, Veracruz, Mexico; 4 Ventura Botanical Garden, Ventura, CA USA

**Keywords:** Natural variation in plants, Plant physiology, Agriculture, Agroecology, Conservation biology

## Abstract

Many parts of the world face severe and prolonged drought conditions, stressing the sustainability of water resources and agriculture. Transitioning to water-efficient crops is one strategy that can help adapt to water scarcity. An emerging drought-tolerant crop of interest is chia (*Salvia hispanica*). Yet, no study has compared its large-scale water use dynamics to those of widely established crops across the globe. Here, we use satellite data over multiple years to assess the water use efficiency of chia, alfalfa, corn, and soybean globally. Results show that chia consumed 13-38% less water than alfalfa, corn, and soy and assimilated 14-20% more carbon per amount of water used. Substituting 10% of Southwest United States alfalfa cultivation with chia would save 184 million liters of water per growing season, equivalent to the annual water consumption of 1,300 households. Future research shall explore the economic, societal, and environmental ramifications of substituting alfalfa with chia in dry areas worldwide. These insights can guide decision-makers in promoting sustainable agriculture and water resource management.

## Abstract (web summary)

A large-scale remote sensing study on water use by chia suggests that chia could be a sustainable choice for agriculture, consuming 13-38% less water than alfalfa, corn, and soybean, while assimilating 14-20% more carbon per unit of water.

## Introduction

Critical changes are needed to adapt agriculture to increasingly variable and decreasing water resources. The Food and Agriculture Organization estimates that agriculture accounts for 70% of global water use^1^. California’s Central Valley in the Southwest United States (SW-US) accounts for an estimated 8% of the US agricultural output^2^. Historically a fertile breadbasket of the world, over the past two decades, the SW-US has been experiencing the worst drought period in the last 1200 years^3^. Such droughts are increasing in frequency, intensity, and magnitude throughout the world^4^. This poses a threat to the food security of millions of people^5^.

If agriculture is to be sustainable, we must find a balance between preserving the long-term viability of the water supply and ensuring food security. Solving this enormous challenge will require proper crop management strategies, implementation of advanced technologies to help farmers optimize water use, and government policies incentivizing farmers to conserve water^6^. One promising approach to resolving this alarming problem is substituting some portions of the high-water-demanding crops with more water-efficient crop alternatives^7^.

Crop replacement aims to supplant widely established crops with novel or regionally uncommon crops that address a local problem without drastically reducing agricultural output or nutritional quality^7^. Similarly, crops may be replaced with other varietals or genetically modified variants of pre-existing crops to increase output or pest resistance^8^. Often, but not always, these crop replacement endeavors pose tradeoffs to farmers and the agricultural sector, such as increasing food production at the cost of greater resource use during cultivation or increasing economic profitability while reducing food supplies^8^. In this study, we focused on three crops (alfalfa, corn, and soybean) with relatively widespread cultivation and significant importance to target for potential replacement with chia with a case study in the SW-US^9^. The findings from this study could elucidate the potential of chia as a substitute for water-intensive crops in drought-affected areas across the globe. This is particularly important as recent studies have underscored the anticipated escalation in the severity, frequency, duration, and spatial extent of future droughts due to global warming^10^.

Alfalfa is a highly water-intensive and widespread crop, sparking controversy as water supplies diminish^11,12^. Thirty-seven percent of the water from the Colorado River basin (1.97 trillion liters) goes toward growing alfalfa and hay used primarily to sustain a multi-billion-dollar livestock industry in the SW-US^13^ and other parts of the world. Compounding this demand, the Colorado River streamflow has decreased by 16% over the past two decades^14,15^. Replacing some of the alfalfa cultivation with more water-efficient crops is a potential approach to sustainability through water conservation. The strategic replacement of alfalfa could yield substantial benefits in global water conservation, given that over 30 million hectares of alfalfa are currently being cultivated worldwide^16^.

Chia (*Salvia hispanica*) is an emerging seed crop, particularly rich in polyunsaturated fatty acids, mainly alpha-linolenic fatty acid (also known as omega-3 fatty acid or fish oil), dietary fiber, protein, antioxidants, and minerals^17^. Notably, chia can be cultivated with irrigation in dry regions of the world including Chile, Venezuela, Brazil, Argentina, Panama, Costa Rica, Dominican Republic, Honduras, Namibia, South Africa, Tanzania, Democratic Republic of Congo, Uganda, Yemen, Indonesia, Papua New Guinea, India, Vietnam, Kenya, Australia and the SW-US^18,19^. Despite being the largest importer of chia seeds, the commercial cultivation of chia on a large scale is not prevalent in the United States. In a previous research study, we analyzed historical temperature and precipitation data specific to the U.S. to assess the potential for widespread chia cultivation^18^. Our findings indicated that temperature, particularly the occurrence of the first freeze of the year, serves as the primary limiting factor for chia cultivation across the country. As we consider prospective scenarios of a warmer climate in the future, it is anticipated that suitable areas for chia cultivation will expand. Although grown mainly for human consumption, chia seeds can also be used as a nutrient supplement in animal feed^20^ and have improved egg and meat production in poultry and milk production in cows^21^. Moreover, the non-seed (leaf and stem) chia plant is also usable for high-quality livestock feed^22,23^.

Plant breeding is also a critical strategy that aids crop replacement and improves outcomes. By breeding in advantageous traits, plant breeding can increase the benefits and reduce the tradeoffs of crop replacement^8^. Crop breeding has contributed to significant increases in cereal crop yield since 1960, particularly in Europe and North America^24,25^. Chia has a genome nearly an order of magnitude smaller than common crops such as alfalfa, corn, and wheat. This allows for far more rapid genomic selection to improve its agronomic and nutritional qualities than many more established food crops^26,27^. Accordingly, breeding programs may be able to improve chia to meet arising obstacles more quickly than crops with more complex genomes. This trait further contributes to chia’s potential.

Remote sensing via satellite imagery has become a fast and effective approach for measuring and mapping the environment and the ecosystem at different spatial and temporal dimensions^4,28^. These remotely generated data have been used to answer important questions in the fields of ecology, biodiversity conservation, and agriculture and inform policymakers^29,30^. Within the agriculture industry, satellite data has been successfully used in crop type identification, land use, disease or nutrient management, and estimation of water loss due to soil evaporation and plant transpiration (collectively named evapotranspiration or ET)^4,30–34^. Monitoring crop water usage through ET is of paramount importance, especially under conditions of water scarcity^4,34^. While this study leverages remote sensing data from SW-US, Mexico, and Australia, the primary findings potentially apply to other drought-affected regions worldwide. Moreover, the proposed methodology can also be adapted to evaluate the performance of distinct crop species within the unique cultural and economic contexts of various drought-prone areas globally.

NASA’s ECOsystem Spaceborne Thermal Radiometer Experiment on Space Station (ECOSTRESS), launched to the International Space Station in 2018, estimates evapotranspiration (ET) and water use efficiency (WUE), among other variables at 70 m spatial resolution every 1–5 days with good accuracy, especially for agricultural areas^35,36^. Landsat also provides reliable ET data, albeit less frequently than ECOSTRESS, but with a longer record spanning decades^37^. This reliability allows for accurate measurements of water consumption and loss over vast and disparate crop fields where in situ measurements would not be feasible or would be prohibitively expensive. This allows for the collection and analysis of vast amounts of data over large temporal and spatial scales, granting opportunities to study agricultural water resources at much larger scales than would otherwise be possible.

Remote sensing accordingly offers a great boon to crop replacement assessments. By providing large amounts of data, including ET, temperature, and precipitation, extensive comparisons of different crops can be performed worldwide. With such far-reaching analyses—including the capability to analyze numerous sites over vast distances and multiple years—the tradeoffs and benefits of crop replacement initiatives can be understood better before implementation. This gives farmers and other stakeholders a better understanding of what they stand to gain and the potential risks they would encounter before investing in the replacement.

**Fig. 1:**
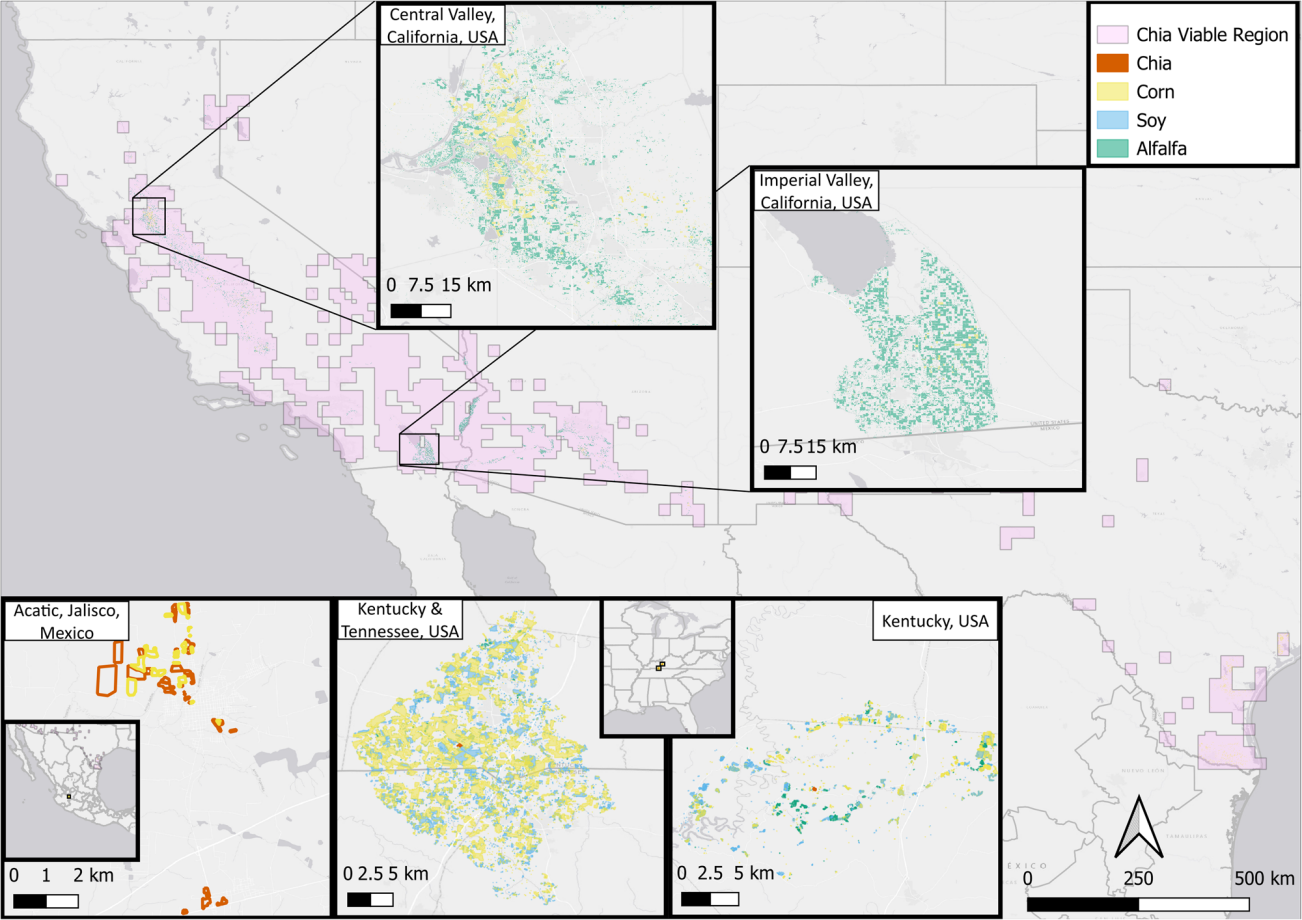
The distribution of the main crops in areas suitable for chia cultivation. Corn (yellow), alfalfa (green), and soybean (turquoise) are among the most prominently cultivated crops near known chia sites (red) in the United States and Mexico and the chia-viable region of the Southwest United States designated by the purple color. Background image from Google Maps.

Insights gained from remote sensing can be combined with ground and laboratory data to synergize data for crop replacement studies. Experimental data regarding the viability of a given crop under different climatic and meteorological conditions can be aided by the analysis of vast quantities of remotely sensed climate and weather data to map out where a crop is most likely to be cultivated successfully^38^. This analysis is of utmost value for crop replacement initiatives by providing a direct understanding of where a replacement crop can be successfully grown and thereby determining how many other crops in which locations can be converted. Through such a study, the regions of the United States where chia can most viably be potentially grown are presented (^18^ and Fig. 1). With a significant swath of the SW-US viable for chia cultivation, including a large portion of the agricultural Central Valley in California, such hybrid remote sensing and direct observation further emphasizes the potential that chia has for crop replacement initiatives. Beyond the regions utilized in this study, chia cultivation could extend to other drought-affected regions worldwide^19^, highlighting its practical implications for global water conservation efforts in arid areas.

In this study, we evaluated total ET, vegetation transpiration, and WUE of chia, alfalfa, corn, and soybean in different locations throughout the world (i.e., North and Central America, Australia) over multiple years (2013–2014 and 2021–2022) using satellite images collected at different times. Results from over 2000 agricultural fields representing 20,761 hectares and 42,000 image pixels showed that chia consumed 13–38% less water than alfalfa, corn, and soybean and assimilated 14–20% more carbon per amount of water used, cumulatively throughout the mean Northern Hemisphere growing season, from late May to early November. We show that replacing just 10% of the current alfalfa cultivation in the SW-US with chia would save 184 million liters of water in a growing season, equivalent to the annual water consumption of 1300 households. Future research should focus on examining the economic, societal, and environmental ramifications of substituting alfalfa with chia in dry areas around the world, which can provide the necessary insights for decision-makers to incentivize the adoption of these crop replacements, ultimately advancing sustainability goals in agriculture and water resources.

## Results

### Understanding water efficiencies

**Fig. 2:**
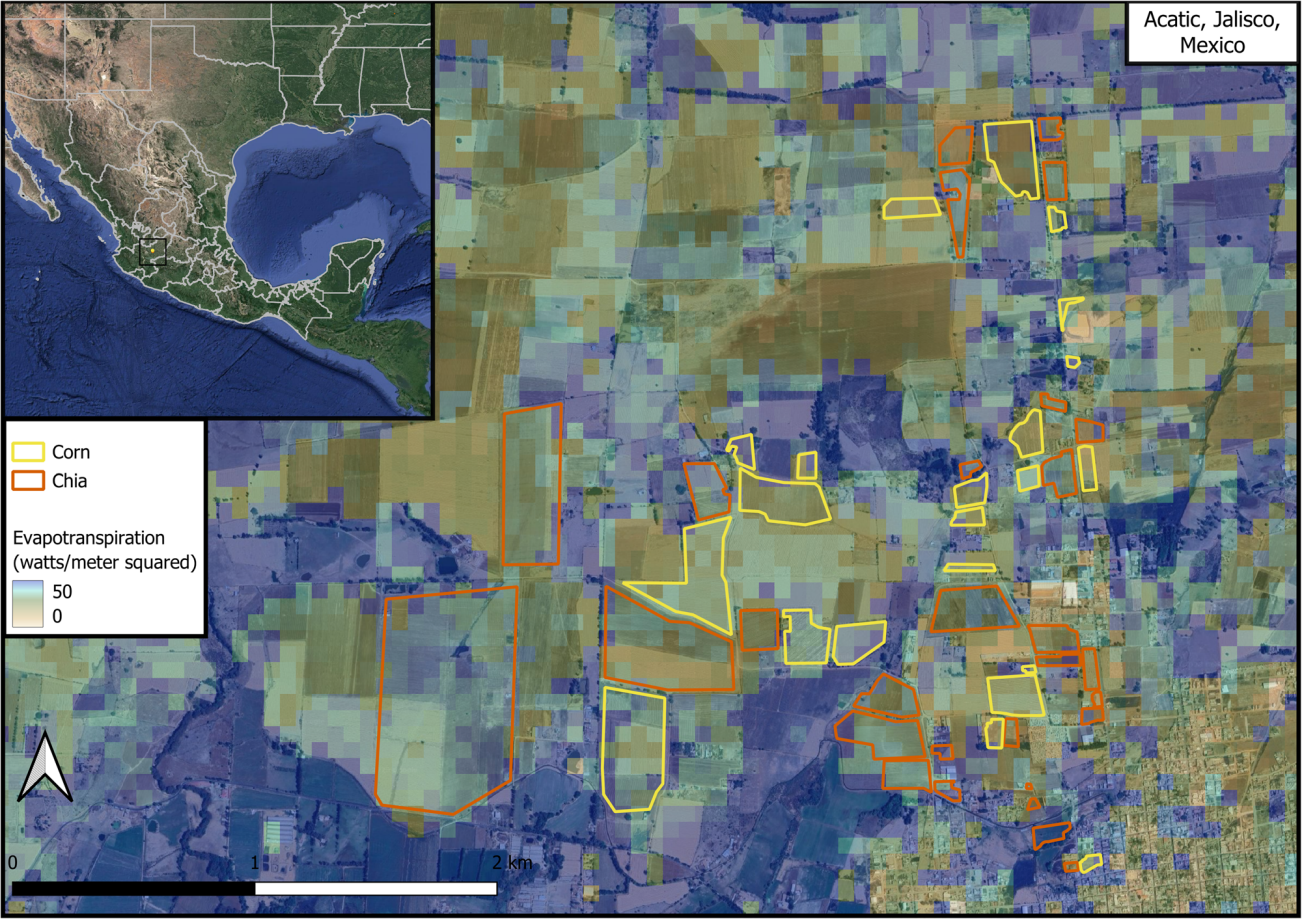
ECOSTRESS provides high-resolution observations of water consumption within and across agricultural fields. The mean daily ET data from May 22, 2022, obtained from ECOSTRESS, is overlaid on corn and chia fields. Yellow and orange boundaries represent corn and chia fields, respectively. Colors represent ET from high (blue) to low (beige). ECOSTRESS data are shown only for our study area. Background image from Google Maps.

**Fig. 3:**
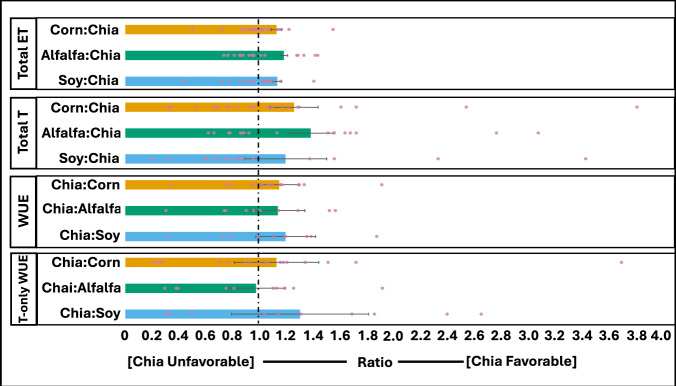
Chia is more efficient than corn, alfalfa, and soy for all water use categories except transpiration-only water use efficiency. Corn and alfalfa’s transpiration and all crops’ evapotranspiration were favorable for chia within the parameter’s standard error range. The data shown are averages across all fields analyzed for chia (*n* = 40), alfalfa (*n* = 130), corn (*n* = 892), and soybean (*n* = 815). Error bars signify the standard error. Evapotranspiration and transpiration represent a ratio of crop to chia, where values greater than 1 signify less water used by chia. Water use efficiency and transpiration-only water use efficiency represent chia-to-crop ratios where values greater than one indicate that chia sequesters more carbon per quantity of water.

Chia consumed less water than alfalfa, corn, and soybean cumulatively throughout the growing season, from late May to early November. Chia sites were located in Kentucky USA, Acatic Mexico, and Western Australia, with one site cultivated throughout two growing seasons (Figs. 1, 2, Supplementary Fig. 1). The total evapotranspiration of chia was on average less than alfalfa (18.3%, SE = 0.029), corn (13.0%, SE = 0.041), and soy (13.4%, SE = 0.035) (Fig. 3). When removing soil evaporation and considering only the growing season’s total transpiration, chia consumed on average even less water than alfalfa (38.5%, SE = 0.17), corn (26.2%, SE = 0.18), and soy (19.6%, SE = 0.31) (Fig. 3).

On average, chia also assimilates more carbon per liter of water consumed, with a higher WUE than alfalfa (14.2%, SE = 0.20), corn (15.2%, SE = 0.15), and soybean (20.0%, SE = 0.22) (Fig. 3). This indicates that chia produces more biomass with the same amount of water than all three crops. When removing soil evaporation and considering transpiration-only WUE, chia averages higher than corn (12.7%, SE = 0.32) and soybean (30.5%, SE = 0.22) but slightly lower than alfalfa (2.9%, SE = 0.51) (Fig. 3).

On average, chia consumed more water per day than alfalfa (1.4%, SE = 0.051), corn (1.8%, SE = 0.030), and soybean (6.2%, SE = 0.026), as measured through evapotranspiration. However, accounting for transpiration alone, chia consumed less water per day on average than alfalfa (23.4%, SE = 0.16), corn (15.0%, SE = 0.18), and soy (5.2%, SE = 0.27). Moreover, chia was found to have a shorter growing season than corn, alfalfa, and soybean in three of the four sites. This shorter growing season compensated for the higher daily ET and compounded with the lower daily transpiration to improve the season totals relative to daily usage.

Chia consumed even less water when analyzing transpiration only. Chia’s WUE was less uniform but still higher than the other crops in most scenarios (Fig. 3). While chia’s transpiration-only WUE was lower than alfalfa’s, chia transpired less than two-thirds as much as alfalfa did in a season, on average, creating a tradeoff of slightly lower carbon sequestration but significantly lower water consumption as well. While chia was irrigated with more water per day, the crop transpired less than each of the other three crops. This indicates the discrepancy may potentially be caused by inefficient irrigation in chia fields due to significantly higher ET than transpiration. If this is, in fact, the case, increased efficiency of irrigation strategies may lead to water usage ratios trending towards transpiration-only values.

### More chia, less water consumed

**Fig. 4:**
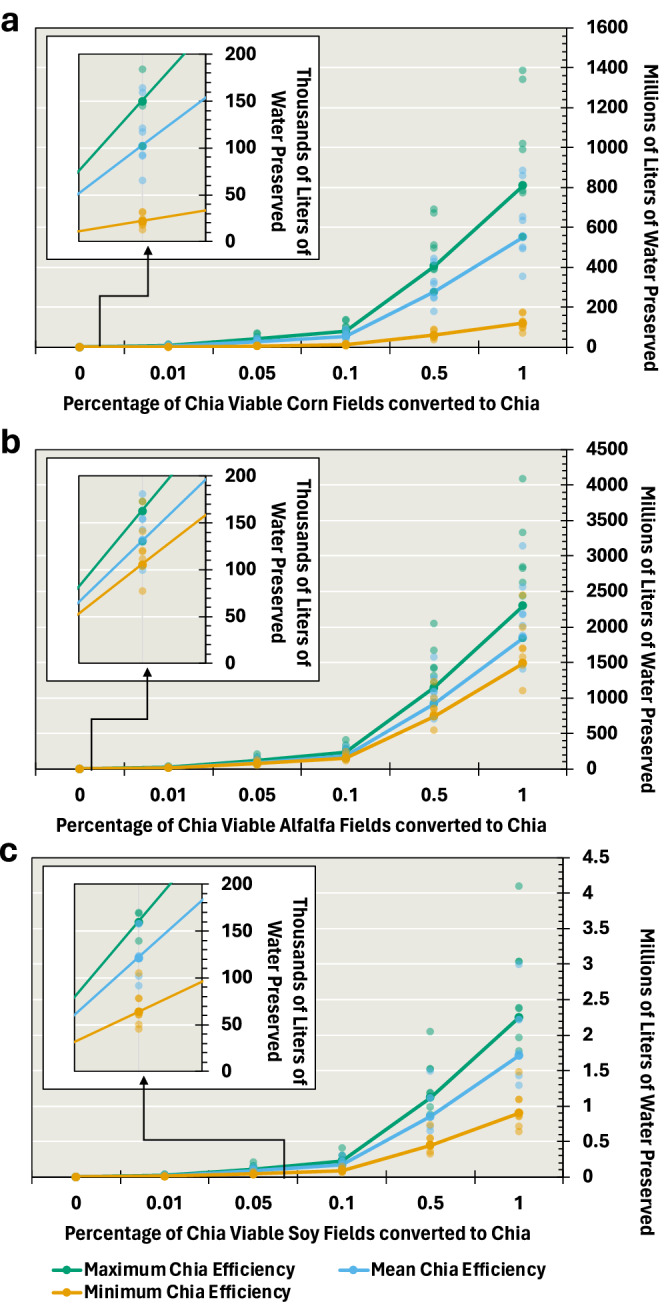
Best-, average-, and worst- water usage scenarios demonstrate water savings when converting to chia cultivation. Crops compared to chia are corn (**a**), alfalfa (**b**), and soybean (**c**). Water usage reduction is calculated with evapotranspiration. Maximum, minimum, and mean are derived as projections from an average across all fields considered for (**a**–**c**) chia (*n* = 40), (**a**) corn (*n* = 892), (**b**) alfalfa (*n* = 130), and (**c**) soybean (*n* = 815).

In regions of the SW-US viable for chia cultivation, there were 14,100 hectares of alfalfa, 5390 hectares of corn, and 14 hectares of soybean cultivated in 2022. While soybean constitutes only a small portion of the cropland cultivated in the SW-US, it is still widely grown in other areas alongside chia (e.g., South America, Australia, China, and India), such that analysis of soybean offers both information regarding crop replacement in other regions and a bridge for comparing chia to other crops using a widely studied crop. With chia consuming less water than the other crops throughout a growing season, converting some of these fields to cultivate chia could reduce agricultural water usage. In aggregation for this study area, replacing crops with chia could preserve an average of 130,900 liters per hectare of alfalfa, 102,400 liters per hectare of corn, and 121,800 liters per hectare of soybean each year, for a total of 355,100 liters of water across all crops considered (Fig. 4, Supplementary Table 1). Even replacing just 10% of each crop’s fields in chia viable areas would save an average of 184,600,000 liters for alfalfa, 55,200,000 liters for corn, and 170,900 liters for soybean, for a total of 239,971,000 liters of water across all crops (Fig. 4, Supplementary Table 2).

The results become more variable when soil evaporation is removed, and these totals are derived from growing season transpiration totals. However, there are still positive water savings in all but the lowest-savings scenarios for corn and soybean (Supplementary Fig. 2). Each hectare converted to chia preserves an average of 180% of the ET-based value for alfalfa, 57% for corn, and 140% for soybean. While corn has reduced average savings and both corn and soybean have negative scenarios, the average water savings for alfalfa and soybean are significantly higher than ET indicates. When converting 10% of each crop to chia, water savings can reach up to 350% of the ET-based combined savings.

No statistically significant relationships were found between temperature and the performance of chia versus other crops in ET, transpiration, WUE, or transpiration-only WUE (Supplementary Fig. 3; Supplementary Table 3). As such, temperature does not appear to directly impact the relative advantages of chia over corn, soybean, and alfalfa, thereby providing climatic robustness in viable areas. The daily and seasonal average differences between chia and these other crops should be consistent without being solely driven by the temperature of the area in which they are cultivated.

With ET and transpiration-based analyses, converting existing corn, alfalfa, and soybean cropland to chia presents the opportunity to preserve tens of thousands to millions of liters of water without sacrificing carbon gain and production. There is even further potential to replace other crops, such as walnuts, citrus, pistachios, and almonds (Supplementary Fig. 4). Alfalfa presents both the highest per hectare and highest per percentage savings when converted to chia, both with ET and transpiration-only calculations.

## Discussion

Overall, our results show that chia consumes 13–18% less water compared to alfalfa, corn, and soybean over the growing season (Fig. 3). Moreover, chia produces 14–20% more biomass per liter of water consumed, making it a more water-efficient crop-per-drop than alfalfa, corn, and soybean. In terms of water savings, replacing alfalfa would conserve an average of 130,900 liters of water per hectare (Fig. 4). Still, among the perennial leguminous forage crops, alfalfa is the most widely utilized in the livestock industry in the United States as hay^39^. The combination of high vegetative biomass yield, nutritional quality, adaptability to the environment, perennial growth, soil improvement qualities, ability to fix atmospheric nitrogen with the help of symbiotic nitrogen-fixing bacteria, and other positive attributes have made alfalfa a staple in animal agriculture^40^.

Nonetheless, given the widespread alfalfa production in areas suitable for chia cultivation in the SW-US, replacing just 10% of the current alfalfa cultivation with chia would save 184 million liters of water within 5 months of the growing season. This is equivalent to the annual water consumption of 1,300 households. Thus, chia could help alleviate some of the future negative impacts of water scarcity in the region. Our previous research conclusively demonstrated that cold temperatures, specifically the timing of the end-of-season freeze, constitute the primary limiting factor for widespread chia cultivation in the United States. This conclusion aligns with the findings by ref. ^19^. Analyzing over a century of data collected from weather stations, researchers observe that the first freeze of the year has been occurring later in the calendar both in the United States and worldwide^41,42^. This temporal shift has positive and negative implications, depending on the specific crop under consideration. Another critical aspect to consider is the interplay between warming temperatures and rainfall patterns. Climate models consistently predict increased atmospheric water vapor as the planet warms. However, recent research has revealed that atmospheric moisture over arid and semi-arid regions has not risen as expected in the context of global warming^43^.

Consequently, given the combination of the delayed appearance of the first freeze and the lack of increase in late-summer precipitation, we cautiously anticipate that climate change will not significantly affect chia cultivation. This resilience to climate variations reinforces the suitability of chia cultivation in regions where it thrives. Moreover, climate change may indeed act as a facilitator for chia cultivation, elevating its significance as a strategy to mitigate water scarcity in arid areas, both within the United States and globally. Additionally, our research findings underscore the robustness of chia compared to other crops in terms of ET, transpiration, WUE, and transpiration-only WUE under varying temperature conditions. This suggests that chia could emerge as a resilient choice in the face of evolving environmental circumstances across numerous drought-affected regions worldwide.

The variations in total ET, total transpiration, WUE, and transpiration between the best- and worst-case scenarios of chia’s water efficiency relative to other crops appear to stem from the differences in water consumption calculated across the different years and locations from which data was collected. Less than a 10% difference in ET was observed between consecutive years or locations. However, within the exact location, the variability in ET was less than 5% across consecutive years (Supplementary Table 4). ET represents the combined loss of water from both evaporation from the soil and transpiration from plants^36^. Consequently, different irrigation systems and management practices can influence the rate and efficiency of water use in agricultural settings, ultimately impacting total ET^44^. For instance, drip irrigation will minimize soil evaporative losses relative to flood or sprinkler irrigation, providing water directly to the root zone^45–47^. This partly explains the observed variability in our ET calculations, which can be reduced by controlling for irrigation efficiency and uniformity in future research. Furthermore, the absence of widespread commercial cultivation of chia in the United States limited our ability to increase the sample size, which could also help reduce the variability and improve the precision of the measurements. Despite the observed variability in WUE among each of the four crops analyzed in this study, chia is a more water-efficient crop on average.

As a crop that re-emerged relatively recently, chia has been the subject of only limited experimentation. However, due to its recognized potential, we anticipate that chia will undergo extensive study in the future. The lack of widespread commercial cultivation of chia in the United States currently impedes our ability to thoroughly investigate the potential impact of climate change on its WUE and transpiration using historical data. Future research that correlates warmer temperatures with chia transpiration and WUE, compared to other crops, will further underscore the resilience of chia under anticipated climate change scenarios.

Adapting to the challenges posed by climate change in agriculture requires strategic rotations of crop varieties and species. This ongoing process of changing and diversifying crop varieties is essential to ensure that agriculture remains resilient in the face of constantly shifting environmental conditions^8^. Although crop replacement could provide viable agroecological solutions, several economic, policy, and social factors may affect growers’ willingness to change their cropping practices^48^. Efforts should be focused on regions with severe water scarcity and high agricultural demand. These areas may include regions facing acute drought conditions or where underground water sources are running dry.

Moreover, factors such as crop water requirements, local climate, and soil conditions should be considered and studied before large-scale replacement initiatives. The proportion of crop replacement will vary based on local contexts, such as assessments of water availability, crop yield potential, and economic impact, to determine an appropriate balance. Altogether, effective water conservation through crop replacement requires a holistic approach, including soil management, irrigation practices, and community engagement. Finally, it is essential to recognize that even though we have demonstrated chia’s improved water efficiency compared to alfalfa, the practical replacement of alfalfa with chia should be supported by further research on chia’s vegetative parts for livestock feed.

To date, the utilization of chia in animal nutrition has been limited to the consumption of its seeds. Chia seeds have been successfully utilized in poultry, ruminants for meat and milk production, rabbit, pork, fish, and even edible insects^21^. While research on the utilization of chia vegetative biomass remains limited relative to alfalfa, corn, and soybean, recent studies have yielded encouraging findings regarding its potential as a livestock feed source. The highest quality forage for chia can be obtained by harvesting plants at the early vegetative stage^23^. In addition to meeting the forage crop standards set by alfalfa, chia holds potential advantages due to the potential high omega-3 unsaturated fatty acid content in its vegetative parts. Currently, one of the main goals of the dairy and meat industry is to improve omega-3 content. Saturated fatty acids like omega-6 are prevalent in the diets of both animals and humans in Western countries. A high intake of saturated fatty acids has been linked to various health issues, including cardiovascular diseases, diabetes, obesity, and high blood pressure^49–51^. Consequently, forage with a low omega-6/omega-3 ratio is regarded as a significant factor in promoting healthier animal food choices. The chia omega-6/omega-3 ratio in early to late vegetative stages ranges between 0.17 and 0.26, which is lower than alfalfa (0.59), making chia a more nutrient-rich alternative^23^.

Another critical component of forage quality is digestibility, which is still a primary bottleneck in alfalfa, and there is considerable interest in improving alfalfa digestibility. According to Castler and Vogel (1999)^52^, if there were a 1% improvement in the digestibility of forage, it is estimated that this enhancement would lead to a substantial 3.2% increase in the daily weight gain of beef steers. Unlike the remarkable success of plant breeding in diploid crops, improvements in outcrossing polyploid crops (such as alfalfa) have lagged mainly due to the complexity of their genome composition and breeding. As such, progress in developing alfalfa cultivars with improved digestibility has been slow due to the quantitative nature of the trait, tetraploid genome, and large genome size (2738 Mb)^53,54^. Still, recurrent selection for alfalfa stem in vitro neutral detergent fiber digestibility approach showed potential for improving alfalfa stem digestibility^55^.

Despite these promising advancements in improving alfalfa digestibility, the process is very slow and challenging. On the other hand, chia has a relatively much smaller genome (347 Mb) with only six chromosomes in a haploid state^56^. This represents a major advantage, as chia’s compact genome makes it conducive to rapid genetic improvements of its agronomic and nutritional qualities through plant breeding. Hence, even though chia has not been subjected to the same level of extensive research as alfalfa, future breeding endeavors to enhance its nutritional attributes as forage for livestock are anticipated to bridge the gap with and even exceed alfalfa within a relatively brief period.

An important consideration for crop replacement, in addition to climate resilience, water savings, and nutrition, is the overall economic benefits necessitating some rough order of magnitude estimates to start. Here, approximate calculations were carried out to estimate potential financial gains or losses that individual farmers might experience when replacing one hectare of corn, alfalfa, or soybean with chia in the SW-US. This inquiry aimed not to determine a definitive monetary value but to begin theorizing, as a thought exercise, about potential scenarios and trends. We calculated potential monetary impacts by analyzing a host of crop yields and sale prices, as well as a water price from California’s Central Valley and the comparative water usage determined above. Due to the wide-ranging prices and the variation in observed water usage, the monetary impact varied between a 90% loss and a 3000% gain. However, the mean impact from replacing corn, alfalfa, and soybean with chia was a roughly $10,000 increase per hectare replaced—an increase of 300–1000% (Supplementary Fig. 5). While these economic estimates should be taken very lightly due to the uncertainty of values and unknown costs associated with converting fields or changes to labor or equipment, they positively indicate a possible material benefit beyond environmental concerns.

The demand for chia has steadily increased since 2005, when chia was recognized as a food for the first time in the United States. As a result, the United States has become the world’s largest importer of chia seeds, with the value of imported chia seeds reaching $184 million in 2022^57^. The demand for chia has recorded a 2.4% Compound Annual Growth Rate (CAGR) between 2018 and 2022. According to Future Market Insights (FMI), chia demand is predicted to record a 7% CAGR from 2023 to 2033^58^. Altogether, chia cultivation could be a profitable alternative to some commonly cultivated crops. With an already-increasing trajectory of adoption based on economic projections, climate change should only add to this acceleration.

**Fig. 5:**
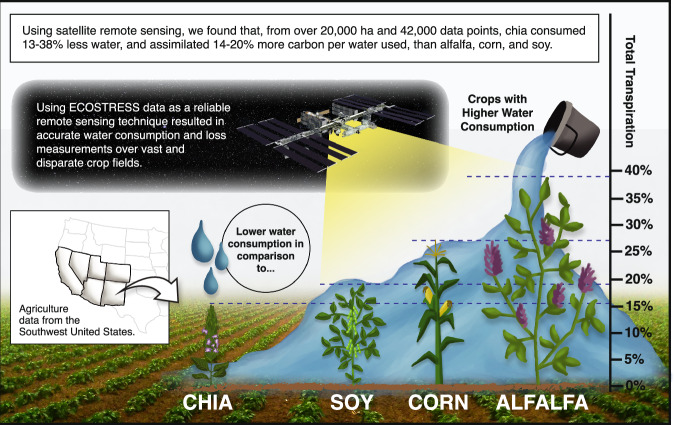
There is a potential of 13–38% water savings through crop replacement in the southwest United States, from alfalfa, corn, and soy to chia.

In summary, balancing sustainability in water resources while ensuring food security amid a growing population presents a formidable challenge. Addressing this challenge requires a multifaceted approach encompassing economic strategies, conservation efforts, and effective policy measures. In our study, we demonstrate that substituting a portion of the water-intensive alfalfa cultivation with chia could lead to substantial water savings (Fig. 5). The research findings underscore chia’s potential as a viable alternative to alfalfa in the arid regions of the world such as the SW-US. Results indicate that chia consumed 13–38% less water than alfalfa, corn, and soybean and assimilated 14–20% more carbon per amount of water used, cumulatively throughout the mean Northern Hemisphere growing season, from late May to early November. Implications for water savings can be demonstrated by replacing just 10% of the current alfalfa cultivation in the SW-US with chia. This would result in 184 million liters of water in a growing season, equivalent to the annual water consumption of 1300 households.

Beyond its low water requirements and nutrient-rich seeds, chia also offers the prospect of utilizing its vegetative parts as livestock feed, aligning with the primary objective of alfalfa cultivation. Further research, particularly directly observed or experimental research, would greatly benefit from increased chia cultivation by multiple farmers within the world’s dry regions. Such an expansion of chia cultivation would be deeply valuable for further study and improved confidence in the scope of chia’s benefits relative to other crops. Future research should focus on:

- Analysis of remote sensing data on larger areas with different climates combined with in situ evapotranspiration data.
- Investigating the robustness of chia’s water-saving capabilities under varying levels and durations of drought through multi-location field trials.
- Investigating the effects of agronomic practices on chia biomass production and water use efficiency.
- Breeding for new varieties that produce higher biomass per unit of water used and have higher forage quality that would result in overall high feed intake due to better digestibility in livestock rumen.
- Exploring the possibility of multiple harvests during the season (similar to alfalfa) to obtain high-quality forage.

## Methods

### Locating agricultural fields

To determine the potential water conservation impacts of cultivating *Salvia hispanica* (chia) on a large scale in the SW-US, the water efficiency and usage of chia relative to more widespread crops had first to be determined. Determining these relationships required the study of pre-existing commercial-scale chia cultivation. Chia is cultivated in large quantities in and surrounding the city of Acatic in Jalisco, Mexico (20°46’ N, 102°54’ W). Specific fields growing chia around Acatic were located, and their field boundaries and coordinates were recorded using GPS devices. Chia is also cultivated in Western Australia’s Ord River Irrigation Area in the Kimberley region (15°30′ S, 128°21′ E). The specific coordinates of only one chia field were provided due to privacy concerns. Companies growing and selling chia were contacted to locate additional fields. This rendered the coordinates of a field cultivated in 2013 and 2014 by Roundstone Native Seed (37°22’ N, 85°59’ W) and another cultivated in 2021 by Heartland Chia (36°40’ N, 86°37’ W), both in Kentucky, U.S.A. Representatives from Roundstone Native Seed and Heartland Chia also provided planting and harvest dates for their chia fields. Important crops cultivated near each of these sites were identified, and analysis was conducted on corn, alfalfa, and soy. The sample size for chia was 40 individual fields (350 ECOSTRESS pixels). The sample size for corn was 890 ± 89 fields (28,000 pixels). The sample size for alfalfa was 130 ± 13 fields (730 pixels). The sample size for soy was 820 ± 82 fields (13,000 pixels).

Corn and alfalfa were chosen as the primary crops to compare to chia due to their abundance near these known sites as well as their representation of a significant share of the total cropland within areas in the SW-US potentially viable for chia cultivation^16^. Soybeans were also analyzed due to their proximity to chia sites and their prominence outside of the SW-US, despite a relative infrequency in chia-viable regions of the SW-US. While not directly actionable in the SW-US, insights gained from soybeans can be extended to other areas or employed to develop comparisons with different crops. Precise locations of these three crops and their relative abundance were determined in Kentucky by accessing the U.S. Department of Agriculture’s Cropland Data Layer (CDL) for the relevant years (https://www.nass.usda.gov/Research_and_Science/Cropland/Release/index.php).

In Acatic, only corn was located, and field boundaries and coordinates were recorded using GPS devices. To increase the sample size for the crops in Kentucky, the U.S. Geological Survey’s 12-digit Hydrologic Units (https://pubs.usgs.gov/wsp/wsp2294/), including and adjacent to the known chia sites were used as boundaries within which all corn, soybean, and alfalfa were analyzed. Additionally, the 2022 CDL was used in conjunction with Hassani et al.’s viable regions to identify all corn, alfalfa, and soybeans in areas in the SW-US with the potential for chia cultivation.

### Satellite data collection

For sites active in the 2021 and 2022 seasons, NASA’s Application for Extracting and Exploring Analysis Ready Samples (AppEEARS) was employed to retrieve the daily ECOSTRESS PT-JPL ET, daily ECOSTRESS Water Use Efficiency (WUE), and daily ECOSTRESS Canopy percentage data products (https://appeears.earthdatacloud.nasa.gov/). Due to limits on maximum request size, an in-house Python script ET was used to retrieve daily ECOSTRESS WUE and daily ECOSTRESS Canopy percentage data products across the entire chia viable region of the SW-US for the 2022 growing season (https://github.com/szykozlowski/Auto_Appeears_Requests). For the site active in 2013 and 2014 (before ECOSTRESS launch), the daily Landsat SSEBop ET data product was retrieved from the U.S. Geological Survey (https://espa.cr.usgs.gov/). MODIS daily Gross Primary Production—from both the Aqua and Terra satellites—and Harmonized Landsat and Sentinel-2 Land Surface Reflectance Bands 4 and 5 (Red and Near-Infrared, respectively) were also retrieved for 2013 and 2014 from AppEEARS^59^. Additionally, the daily AIRS Daytime 1° Air Temperature at Surface data product was collected from NASA Giovanni (https://giovanni.gsfc.nasa.gov/12iovanni/).

### Data preparation

QGIS 3.30.3 was employed to clip the CDL layers to the boundaries of the aforementioned hydrologic units in Kentucky and the chia viable regions in the SW-US, as well as to remove pixels not contained within target fields from ECOSTRESS evapotranspiration and transpiration data across the whole potentially viable region. The Semi-Automatic Classification Plugin for QGIS was utilized to generate pixel and area counts for each class within the CDL. This was used to calculate the total area of each crop and the percentage of the region in which each crop is cultivated. We removed non-target pixels from the satellite data collected for the four chia sites. QGIS was then used to generate statistics for the cells in each raster to allow for data analysis. The Semi-Automatic Classification Plugin for QGIS was utilized to generate pixel and area counts for each class within the CDL. This was used to calculate the total area of each crop and the percentage of the region in which each crop is cultivated.

We converted the HTML outputs of QGIS statistics into CSV files for all data collected. These were then filtered for files containing no data and imported into tables in Microsoft Excel through Excel’s power query function.

### Data analysis

For the 2013 and 2014 sites, the Harmonized Landsat and Sentinel 2 Land Surface Reflectance data were used to calculate NDVI. The MODIS Aqua and Terra Gross Primary Production data were also averaged for these sites and divided by ET to generate WUE. Then, for each of the four chia-cultivating sites, each crop’s ET and WUE were multiplied by that day’s canopy percentage to generate transpiration and transpiration-only WUE. For the four chia sites, for each crop, a daily mean for each month was calculated for each parameter to prevent uneven distributions of data from impacting results.

For ET and transpiration, each month’s average was multiplied by the number of days in the month and summated to calculate the total water consumed in each crop’s growing season. The growing season totals for corn, alfalfa, and soybean were each divided by that season’s chia total to generate a crop-to-chia ratio, where higher values indicate less water evaporated and or transpired by chia. The crop-to-chia ratios were then averaged across the four seasons to provide a mean ratio of the water used by corn, alfalfa, and soybean in a growing season compared to that used by chia. Additionally, each month’s mean was also averaged to generate average ET and transpiration values for the entire growing season. The growing season means for corn, alfalfa, and soybean were likewise divided by the averages for chia to generate a crop-to-chia ratio where a higher value indicates that chia evaporates and or transpires less water per day on average during the growing season than the other crops. The crop-to-chia ratios for each season were likewise averaged together to provide a mean ratio across all four growing seasons. Standard error values were calculated from the differences in each season’s ratios. Each individual day’s ET and transpiration ratios were also calculated and averaged to determine the mean daily crop:chia ratios.

For WUE and transpiration-only WUE, each month’s mean was averaged to generate an average ratio of carbon sequestration to water evaporating and/or transpiring during each crop’s growing season. The growing season mean for chia was divided by that season’s corn, alfalfa, and soybean averages to generate a chia-to-crop ratio, where higher values indicated more carbon sequestered by chia per quantity of water. The chia-to-crop ratios were then averaged across the four seasons to provide a mean ratio of the WUE of chia compared to corn, alfalfa, and soybean. Standard error values were calculated from the differences in each season’s ratios. Each individual day’s WUE and transpiration-only WUE ratios were also calculated and averaged to determine the mean daily chia:crop ratios.

For temperature, each day and location were matched with the corresponding data point for each crop at each site to create coordinate points correlating temperature and each parameter for each crop. Linear regressions were derived for each parameter and each crop. Two-tailed t-tests were performed to determine the statistical significance of each crop and each parameter’s linear regression.

### Water consumption in chia-viable areas

For the region of the SW-US potentially viable for chia cultivation, transpiration and ET were averaged for each month to provide each month’s daily mean values. Each month’s average was multiplied by the number of days in that month to give totals, which were summated together to generate seasonal means for each crop. These were then multiplied by the total area cultivating that crop to provide total water ET and transpiration totals for the entire region. These totals were then divided by the crop:chia ratios at different weights to project how much water consumption would be reduced by replacing various amounts of these crops in chia-viable regions with chia. This was calculated using the average crop:chia ratio as well as the minimum and maximum ratios.

### Potential profitability

To provoke discussion, research was conducted to determine a range of per-hectare yields for chia, corn, alfalfa, and soybean. Research was continued to determine a range of prices for the sale of each crop. These were used to calculate the mean, minimum, and maximum gross income provided by harvesting a hectare of each crop. The minimum, mean, and maximum water savings, as estimated through the calculated growing season totals, were multiplied by the price of water for agricultural use in California’s Central Valley (as a stand-in for water prices throughout the chia viable region of the SW-US) to determine how much more would be spent to irrigate each crop relative to chia. These values were subtracted from the gross income per hectare for each crop, such that the highest crop income was matched with the lowest additional price for water relative to chia. This was done to create worst-case, average, and best-case scenarios for chia, where each crop was at its worst performance in the range while chia was at its best and vice versa.

The gross income for chia in the maximum of the range was subtracted by the net income of each crop in the minimum of its range, and the minimum of chia was subtracted by the maximum of each other crop. The resultant figures were a worst-case, average, and best-case scenario of the change in profit for a farmer for each hectare of a crop replaced with chia. These values were multiplied by different weights to provide the change in income when converting different percentages of each crop to chia.

### Statistics and reproducibility

Our research utilized publicly accessible satellite imagery and temperature and precipitation data. The rationale behind our analyses and the specific parameter settings employed are thoroughly described in their respective Methods sections. Supplementary data files offer detailed insights into the calculations and formulas used to derive the results presented in the figures.

### Reporting summary

Further information on research design is available in the Nature Portfolio Reporting Summary linked to this article.

## Supplementary information

Supplemental material

Description of Additional Supplementary Files

Supplementary Data

Reporting Summary

## Data Availability

ECOSTRESS data retrieved from AppEEARS at https://appeears.earthdatacloud.nasa.gov/ Landsat data retrieved from EROS at https://espa.cr.usgs.gov/index/ The source data behind the graphs in the paper can be found in Supplementary Data. Any remaining data supporting this study’s findings are available from the corresponding author upon request.

## Code availability

The code used to retrieve daily ECOSTRESS WUE and daily ECOSTRESS Canopy percentage data is available at 10.5281/zenodo.13529530.
