# Supplementary material for: Satellite observations indicate that chia uses less water than other crops in warm climates
Source: Commun Biol. 2024 Sep 30;7:1225. doi: 10.1038/s42003-024-06841-y (PMC11442738; doi:10.1038/s42003-024-06841-y)
Supplement: Supplementary file 1 — Supplemental material [file 42003_2024_6841_MOESM1_ESM.pdf]

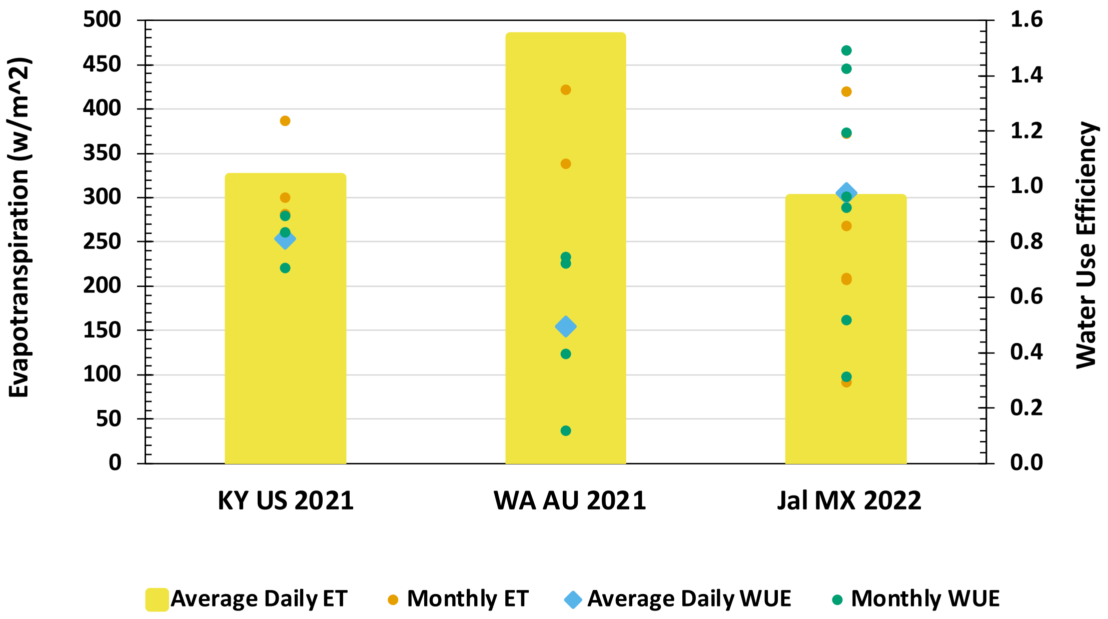

Fig. S1. Chia cultivated in Jalisco, Mexico had lower daily ET and higher daily Water Use Efficiency than in Kentucky, United States, and Western Australia, Australia. The sample size is 37 for Jalisco, 2 for Kentucky, and 1 for Western Australia. Daily average parameters are derived from the growing season of Chia in that location. The results in Kentucky and Jalisco are far more similar to each other than to Western Australia; variance may be driven by differing irrigation strategies.

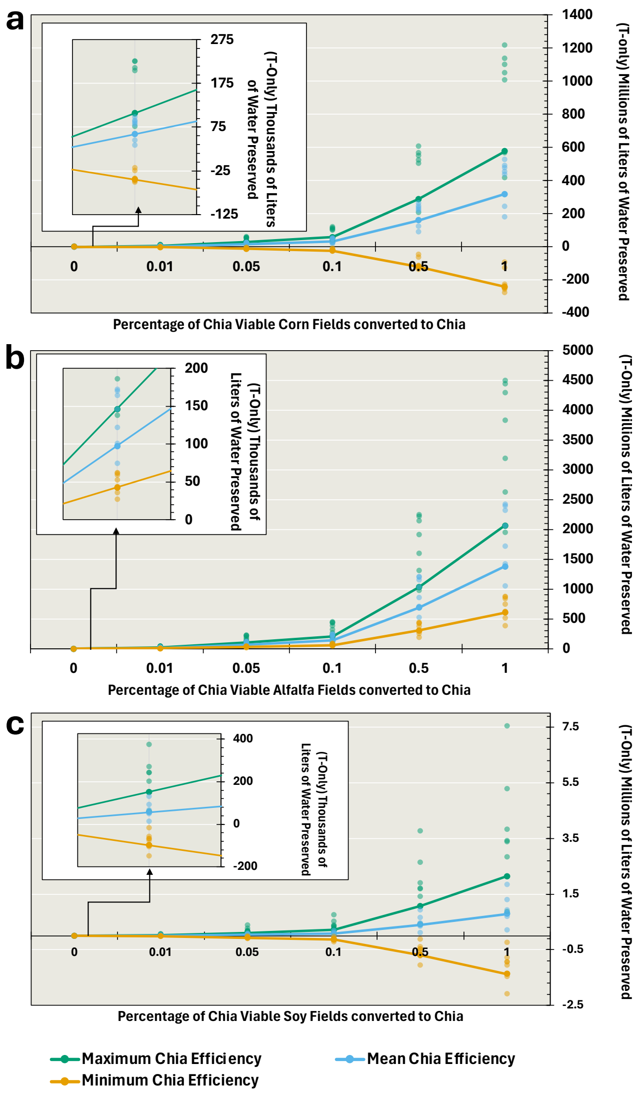

Fig S2: Agricultural water savings when converting to chia cultivation. Crops compared to chia are corn **(a),** alfalfa **(b)**, and soybean **(c)**. Water usage reduction is calculated with transpiration only. Maximum, minimum, and mean are derived from a sample size of 40 for chia projections **(a-c),** 892 for corn projections **(a)**, 130 for alfalfa projections **(b)**, and 815 for soybean projections **(c)**.

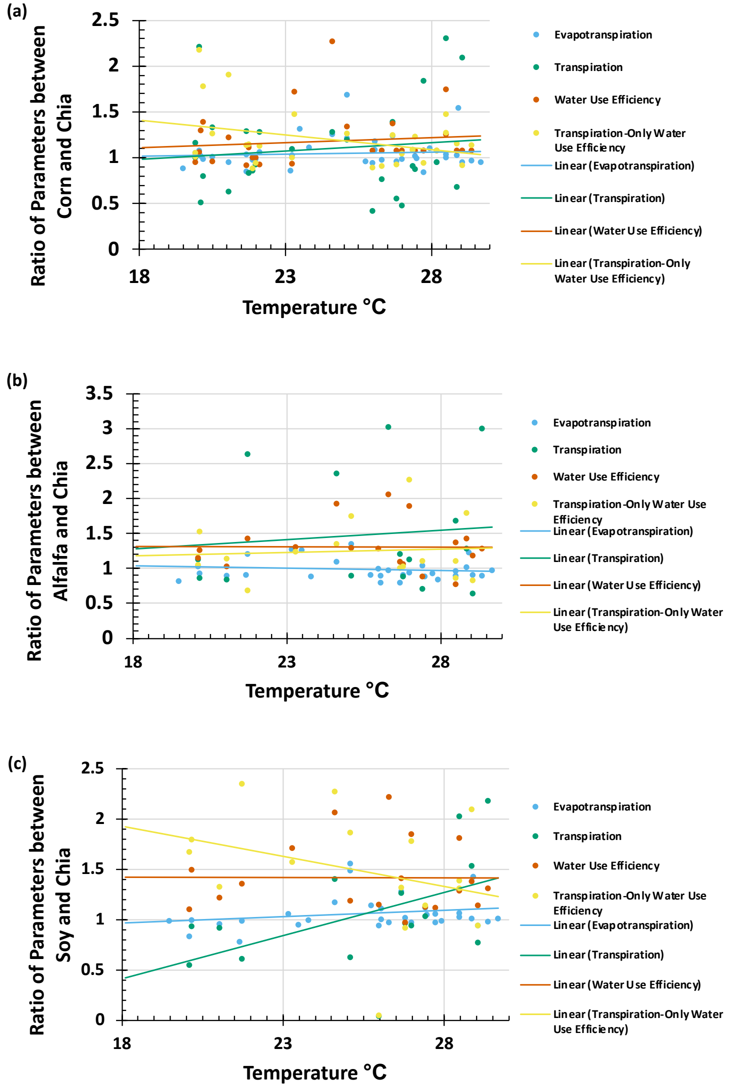

Fig S3: Relationship between daily temperature, in degrees Celsius, and the ratios of evapotranspiration, transpiration, water use efficiency, and transpiration-only water use efficiency between chia and (a) corn, (b) alfalfa, and (c) soybean. Values greater than 1 indicate chia outperforming other crops, and values below 1 indicate chia underperforming other crops. All linear relationships were found to not be statistically significant ($p < 0.05$) and failed to reject the null hypothesis that temperature does not impact the ratio of parameters for chia and other crops.

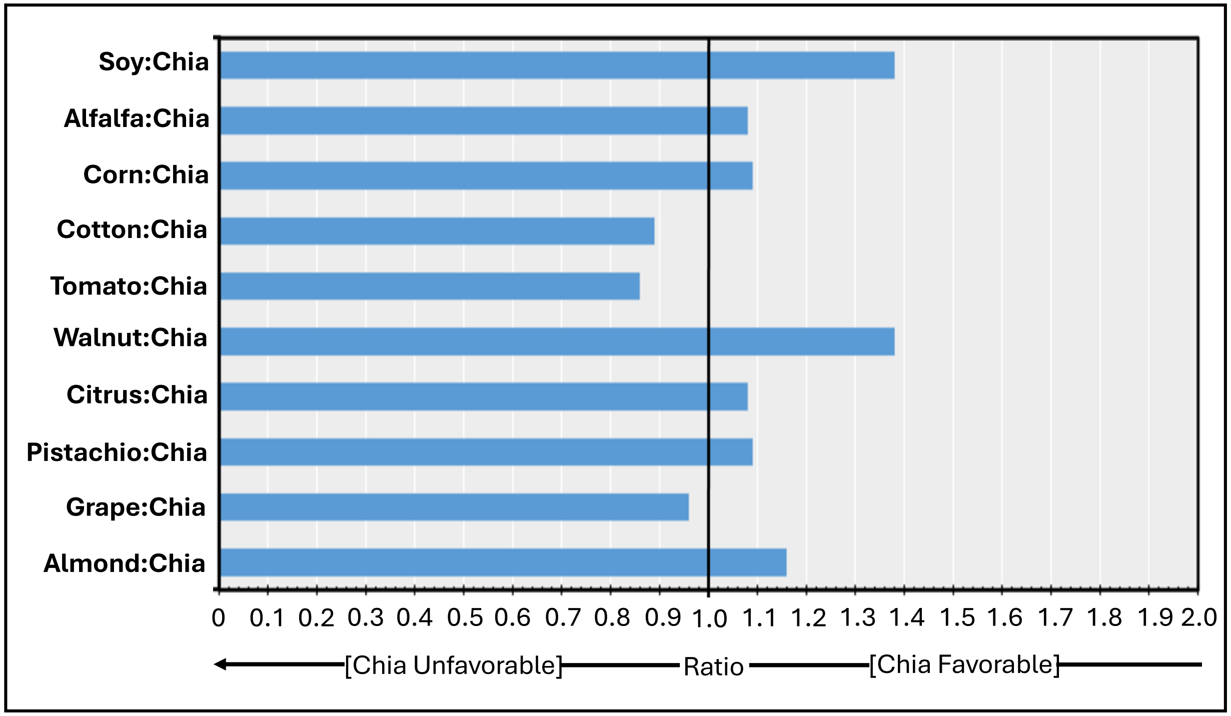

Fig. S4. Chia uses less water during the summer than tree crops such as citrus, walnut, pistachio, and almond in California's Central Valley. By comparing various crops water consumption to the water consumption of corn and alfalfa, chia's consumption can be substituted in to estimate the ratio of evapotranspiration between these crops and chia. Corn, alfalfa, and soy to chia ET ratios from the earlier study provided for comparison.

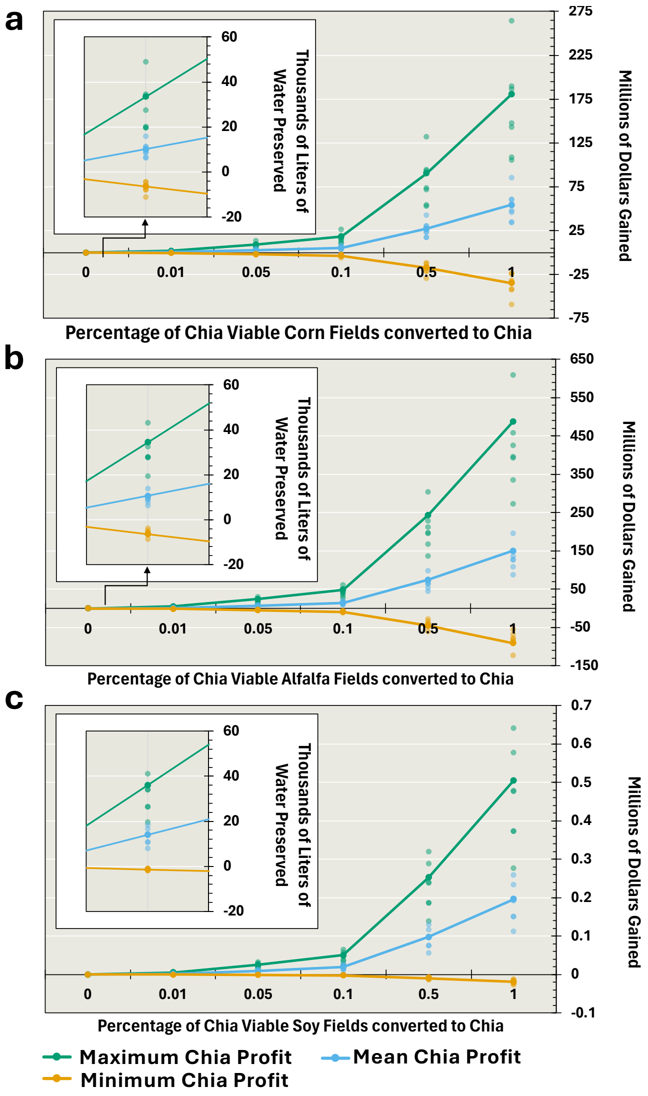

Fig. S5. Replacing significant portions of cropland with chia could provide large increases in profitability to farmers. However, more study is required to increase the accuracy of these estimates.

Table S1: The amount of water savings for each hectare of crop replacement with chia.

Crop	Liters of water preserved per hectare
Alfalfa	130,900 (105,500 - 162,900)
Corn	102, 400 (22,250 - 150,300)
Soybean	121,800 (63,970 - 159,900)
Total	355,100 (191,700 - 473,100)

Table S2: The amount of water savings for 10% of crop replacement with chia.

Crop	Liters of water preserved by replacing 10% of each crop
Alfalfa	184,600,000 (148,800,000 - 229,600,000
Corn	55,200,000 (11,990,000 - 81,020,000)
Soybean	170,900 (89,760 - 224,300)
Total	239,971,000 (160,900,000 - 310,800,000)

Table S3: There is no statistically significant relationship between temperature and evapotranspiration, transpiration, or water use efficiency. A t-test illustrates that p-values for all crop and parameter combinations is above the 0.05 threshold for statistical significance.

	P values of parameters across temperatures by crop		
Crop	Evapotranspiration	Transpiration	Water use efficiency
Alfalfa	0.498	0.709	0.977
Corn	0.564	0.566	0.514
Soybean	0.22	0.057	0.978

Table S4: ET readings varied by less than 10% across consecutive years and locations. 2013 and 2014 data retrieved from Landsat, 2021 and 2022 data collected from ECOSTRESS.

Crop	Evaporation by crop, location, and year			
	KY, 2013 (mm)	KY, 2014 (mm)	KY, 2021 (W/M^2)	Jal, 2022 (W/M^2)
Chia	3046.42	3198.67	322.69	298.807
Alfalfa	3239.14	3220.15	286.86	NA
Corn	3143.97	3101.74	290.704	306.486
Soybean	3010.18	3002.72	289.98	NA
