# Supplementary material for: Satellite observations indicate that chia uses less water than other crops in warm climates
Source: Commun Biol. 2024 Sep 30;7:1225. doi: 10.1038/s42003-024-06841-y (PMC11442738; doi:10.1038/s42003-024-06841-y)
Supplement: Supplementary file 2 — Description of Additional Supplementary Files [file 42003_2024_6841_MOESM2_ESM.pdf]

Description of Additional Supplementary Files

File name: Supplementary Data
Description: The source data of the graphs and charts presented in the main figures.
