# Supplementary material for: Satellite observations indicate that chia uses less water than other crops in warm climates
Source: Commun Biol. 2024 Sep 30;7:1225. doi: 10.1038/s42003-024-06841-y (PMC11442738; doi:10.1038/s42003-024-06841-y)
Supplement: Supplementary file 4 — Reporting Summary [file 42003_2024_6841_MOESM4_ESM.pdf]

nature portfolio

Corresponding author(s): Hagop S. Atamian

Last updated by author(s): Jul 17, 2024

Reporting Summary

Nature Portfolio wishes to improve the reproducibility of the work that we publish. This form provides structure for consistency and transparency in reporting. For further information on Nature Portfolio policies, see our Editorial Policies and the Editorial Policy Checklist.

Statistics

For all statistical analyses, confirm that the following items are present in the figure legend, table legend, main text, or Methods section.

n/a	Confirmed	
☐	☒	The exact sample size (*n*) for each experimental group/condition, given as a discrete number and unit of measurement
☐	☒	A statement on whether measurements were taken from distinct samples or whether the same sample was measured repeatedly
☐	☒	The statistical test(s) used AND whether they are one- or two-sided<br>*Only common tests should be described solely by name; describe more complex techniques in the Methods section.*
☐	☒	A description of all covariates tested
☐	☒	A description of any assumptions or corrections, such as tests of normality and adjustment for multiple comparisons
☐	☒	A full description of the statistical parameters including central tendency (e.g. means) or other basic estimates (e.g. regression coefficient) AND variation (e.g. standard deviation) or associated estimates of uncertainty (e.g. confidence intervals)
☐	☒	For null hypothesis testing, the test statistic (e.g. *F*, *t*, *r*) with confidence intervals, effect sizes, degrees of freedom and *P* value noted<br>*Give P values as exact values whenever suitable.*
☐	☒	For Bayesian analysis, information on the choice of priors and Markov chain Monte Carlo settings
☐	☒	For hierarchical and complex designs, identification of the appropriate level for tests and full reporting of outcomes
☐	☒	Estimates of effect sizes (e.g. Cohen's *d*, Pearson's *r*), indicating how they were calculated

Our web collection on statistics for biologists contains articles on many of the points above.

Software and code

Policy information about availability of computer code

Data collection	Data was collected from publicly available websites following their instructions. One in-house code was used to automate the data acquisition.
Data analysis	QGIS 3.30.3 software was used to prepare the collected satellite images for analysis.

For manuscripts utilizing custom algorithms or software that are central to the research but not yet described in published literature, software must be made available to editors and reviewers. We strongly encourage code deposition in a community repository (e.g. GitHub). See the Nature Portfolio guidelines for submitting code & software for further information.

Data

Policy information about availability of data

All manuscripts must include a data availability statement. This statement should provide the following information, where applicable:

- Accession codes, unique identifiers, or web links for publicly available datasets
- A description of any restrictions on data availability
- For clinical datasets or third party data, please ensure that the statement adheres to our policy

ECOSTRESS data retrieved from AppEEARS at https://appeears.earthdatacloud.nasa.gov/
Landsat data retrieved from EROS at https://espa.cr.usgs.gov/index/
All other data available upon request.

Research involving human participants, their data, or biological material

Policy information about studies with human participants or human data. See also policy information about sex, gender (identity/presentation), and sexual orientation and race, ethnicity and racism.

Reporting on sex and gender	The research findings provided in the submitted manuscript does not involve human participants.
Reporting on race, ethnicity, or other socially relevant groupings	The research findings provided in the submitted manuscript does not involve human participants.
Population characteristics	The research findings provided in the submitted manuscript does not involve human participants.
Recruitment	The research findings provided in the submitted manuscript does not involve human participants.
Ethics oversight	The research findings provided in the submitted manuscript does not involve human participants.

Note that full information on the approval of the study protocol must also be provided in the manuscript.

Field-specific reporting

Please select the one below that is the best fit for your research. If you are not sure, read the appropriate sections before making your selection.

☐ Life sciences ☐ Behavioural & social sciences ☒ Ecological, evolutionary & environmental sciences

For a reference copy of the document with all sections, see nature.com/documents/nr-reporting-summary-flat.pdf

Ecological, evolutionary & environmental sciences study design

All studies must disclose on these points even when the disclosure is negative.

Study description	In this study, we collected satellite images and calculated the water efficiency and usage of chia relative to more widespread crops like corn, alfalfa, and soybean to determine the potential water conservation impacts of cultivating chia on a large scale in the SW-US. The sample size for chia was 40 individual fields (350 ECOSTRESS pixels). The sample size for corn was 890 ± 89 fields (28,000 pixels). The sample size for alfalfa was 130 ± 13 fields (730 pixels). The sample size for soy was 820 ± 82 fields (13,000 pixels). These sample sizes represented all the plots present in the study area. In other words, we did not sample from a population of corn, soybean, chia, and alfalfa plots. We used the whole population in the study area.
Research sample	The population data within the study area was used.
Sampling strategy	No sample size calculation was performed. We used the whole population in the study area.
Data collection	The data used in this study are publicly available. More specifically:<br>ECOSTRESS data was retrieved from AppEEARS at https://appeears.earthdatacloud.nasa.gov/<br>Landsat data was retrieved from EROS at https://espa.cr.usgs.gov/index/
Timing and spatial scale	The daily ECOSTRESS PT-JPL ET, daily ECOSTRESS Water Use Efficiency (WUE), and daily ECOSTRESS Canopy percentage data products were collected for the years 2013, 2014, and 2022.
Data exclusions	No data were excluded from the analysis.
Reproducibility	reproducibility verification was not performed.
Randomization	No randomization was necessary for this study.
Blinding	*Describe the extent of blinding used during data acquisition and analysis. If blinding was not possible, describe why OR explain why blinding was not relevant to your study.*

Did the study involve field work? ☐ Yes ☒ No

Reporting for specific materials, systems and methods

We require information from authors about some types of materials, experimental systems and methods used in many studies. Here, indicate whether each material, system or method listed is relevant to your study. If you are not sure if a list item applies to your research, read the appropriate section before selecting a response.

Materials & experimental systems

n/a	Involved in the study	
☒	☐	Antibodies
☒	☐	Eukaryotic cell lines
☒	☐	Palaeontology and archaeology
☒	☐	Animals and other organisms
☒	☐	Clinical data
☒	☐	Dual use research of concern
☐	☒	Plants

Methods

n/a	Involved in the study	
☒	☐	ChIP-seq
☒	☐	Flow cytometry
☒	☐	MRI-based neuroimaging

Dual use research of concern

Policy information about dual use research of concern

Hazards

Could the accidental, deliberate or reckless misuse of agents or technologies generated in the work, or the application of information presented in the manuscript, pose a threat to:

No	Yes	
☒	☐	Public health
☒	☐	National security
☒	☐	Crops and/or livestock
☒	☐	Ecosystems
☒	☐	Any other significant area

Experiments of concern

Does the work involve any of these experiments of concern:

No	Yes	
☒	☐	Demonstrate how to render a vaccine ineffective
☒	☐	Confer resistance to therapeutically useful antibiotics or antiviral agents
☒	☐	Enhance the virulence of a pathogen or render a nonpathogen virulent
☒	☐	Increase transmissibility of a pathogen
☒	☐	Alter the host range of a pathogen
☒	☐	Enable evasion of diagnostic/detection modalities
☒	☐	Enable the weaponization of a biological agent or toxin
☒	☐	Any other potentially harmful combination of experiments and agents

Plants

Seed stocks	No field experiments were performed as part of this study.
Novel plant genotypes	No novel plant genotypes were produced.
Authentication	No Authentication was needed.
